# Arabidopsis NF–YC7 Interacts with CRY2 and PIF4/5 to Repress Blue Light-Inhibited Hypocotyl Elongation

**DOI:** 10.3390/ijms241512444

**Published:** 2023-08-04

**Authors:** Wei Wang, Lin Gao, Tianliang Zhao, Jiamei Chen, Ting Chen, Wenxiong Lin

**Affiliations:** 1 Fujian Provincial Key Laboratory of Agroecological Processing and Safety Monitoring, College of Life Sciences, Fujian Agriculture and Forestry University, Fuzhou 350002, China; 2 College of Life Sciences, Ningde Normal University, Ningde 352100, China; 3 Key Laboratory of Crop Ecology and Molecular Physiology, Fujian Agriculture and Forestry University, Fuzhou 350002, China; 4 Haixia Institute of Science and Technology, Fujian Agriculture and Forestry University, Fuzhou 350002, China

**Keywords:** NF–YCs, blue light, hypocotyl, Arabidopsis

## Abstract

Light is an important environmental factor. Plants adapt to their light environment by developing the optimal phenotypes. Light-mediated hypocotyl growth is an ideal phenotype for studying how plants respond to light. Thus far, many signaling components in light-mediated hypocotyl growth have been reported. Here, we focused on identifying the transcription factors (TFs) involved in blue light-mediated hypocotyl growth. We analyzed the blue-light-mediated hypocotyl lengths of Arabidopsis TF–overexpressing lines and identified three NF–YC proteins, NF–YC7, NF–YC5, and NF–YC8 (NF–YCs being the short name), as the negative regulators in blue light-inhibited hypocotyl elongation. NF–YC–overexpressing lines developed longer hypocotyls than those of the wild type under blue light, while the deficient mutants *nf*–*yc5nf*–*yc7* and *nf*–*yc7nf*–*yc8* failed to exhibit hypocotyl elongation under blue light. NF–YCs physically interacted with CRY2 (cryptochrome 2) and PIF4/5 (phytochrome interacting factor 4 or 5), while the NF–YCs–PIF4/5 interactions were repressed by CRY2. Moreover, the overexpression of CRY2 or deficiency of PIF4/5 repressed NF–YC7–induced hypocotyl elongation under blue light. Further investigation revealed that NF–YC7 may increase CRY2 degradation and regulate PIF4/5 activities under blue light. Taken together, this study will provide new insight into the mechanism of how blue light inhibits hypocotyl elongation.

## 1. Introduction

Light, an essential and variable environmental factor, regulates the growth and development of plants. Plants have evolved adaptive phenotypes in response to variable light environments (including light quantity, quality, directionality, and photoperiod). For example, Arabidopsis seedlings produce short hypocotyls, de-etiolation, and expanded cotyledons when exposed to light [1].

Plants sense the light environment with light receptors. So far, five kinds of light receptors in Arabidopsis have been found: cryptochromes (CRY1 and CRY2), phytochromes (PHYA–E), phototropins (PHOT1 and PHOT2), three zeitlupe-type receptors (ZTL, LKP, FKF), and a UV–B receptor (UVR8) [2,3,4]. Among them, blue light receptors CRY1 and CRY2 positively regulate blue-light-inhibited hypocotyl growth and long-day-dependent flowering [5,6,7]. CRY2 mainly mediates low-intensity blue-light-induced photomorphogenesis [5]. CRY2 can be activated by light-dependent dimerization and phosphorylation [8,9,10]. Finally, light-activated CRY2 is degraded via ubiquitination [11,12,13].

Many signaling partners such as PIFs, members of the bHLH family, are involved in the CRY–mediated photomorphogenesis [14,15,16]. PIF4 and PIF5 are the light-responsive repressors [14]. The *PIF4/5*-deficient mutants *pif4* and *pif5* showed no obvious hypocotyl growth, compared with the wild type under limiting blue light, while the sustained activation of PIFs led to hypocotyl over-elongation [14]. Therefore, the regulation of PIF activities is necessary for light-mediated hypocotyl growth [14]. CRY2 interacts with PIF4/5 and modulates their activities by occupying the same promoter region as targets that regulate the expressions of light-responsive genes [14]. Additionally, phytochromes antagonize PIF activities by inhibiting the bindings between PIFs and their targets [17,18]. Phosphorylation mediates PIF stabilities. PIFs are inactivated by protein kinases through rapid phosphorylation prior to consequent ubiquitin-mediated degradation [17,18,19,20,21,22]. Light inhibits PIF activities through phosphorylation, ubiquitination, and degradation in a manner dependent on PIF–active phytochrome interactions [23]. Protein phosphatase 6 (PP6) regulates PIF phosphorylation to control the stability of PIFs and their transcriptional activity [24]. 

In plant growth and development, bHLH family members can interact with many signaling molecules, including transcription factor NF–Y (nuclear factor Y containing three subunits: NF–YA, NF–YB, and NF–YC). The NF–YB1–NF–YC12 (nuclear factor transcription factor Y members) heterodimer forms a complex with bHLH144, a member of the bHLH family, to regulate the grain quality in rice [25]. In Arabidopsis, NF–Y transcription factors consist of 10 NF–YA, 13 NF–YB, and 13 NF–YC homologs [26,27]. Based on phylogenetic analyses, the NF–YC family consists of two distinct clades. NF–YC1/2/3/4/9 belongs to one clade, and the second clade contains NF–YC5/6/7/8 and NF–YC10/11/12/13. Siefers et al. analyzed the expression patterns of NF–YC in seedlings [26]. For example, NF–YC1, NF–YC3, NF–YC4, NF–YC9, NF–YC11, and NF–YC12 show hypocotyl expression patterns. Among them, NF–YC1/3/4/9 redundantly promotes photomorphogenesis by light-dependent interactions with HDA15 (histone deacetylase 15), modulating the histone H4 acetylation level and co-repressing the expressions of hypocotyl elongation-related targets [28]. In addition, NF–YC1/3/4/9 positively regulates light-inhibited hypocotyl elongation by interacting with BIN2 (BR-insensitive 2) [29]. Another NF–YC clade member, NF–YC5/7/8, did not show obvious expression patterns in seedlings [26]. 

In this study, we overexpressed Arabidopsis TFs into the background of *rdr6*–*11* (an RNA-dependent polymerase 6 mutant that can repress gene silence) and screened hypocotyl lengths under blue light to identify Arabidopsis TFs involved in blue light-inhibited hypocotyl elongation. In summary, we found that NF–YC5, NF–YC7, and NF–YC8 (NF–YCs being the short name) negatively regulate blue light-inhibited hypocotyl elongation. Moreover, we identified that NF–YC7 interacts with CRY2, PIF4, and PIF5, and CRY2 inhibits NF–YC7–PIF4/5 interactions. NF–YC7 may promote CRY2 degradation and regulate PIF4/5 activities. This work will be helpful for understanding how NF–YC7 suppresses the CRY2 inhibition of hypocotyl elongation under blue light.

## 2. Results

### 2.1. NF–YCs Redundantly and Negatively Regulate Blue Light-Mediated Hypocotyl Growth

**Figure 1:**
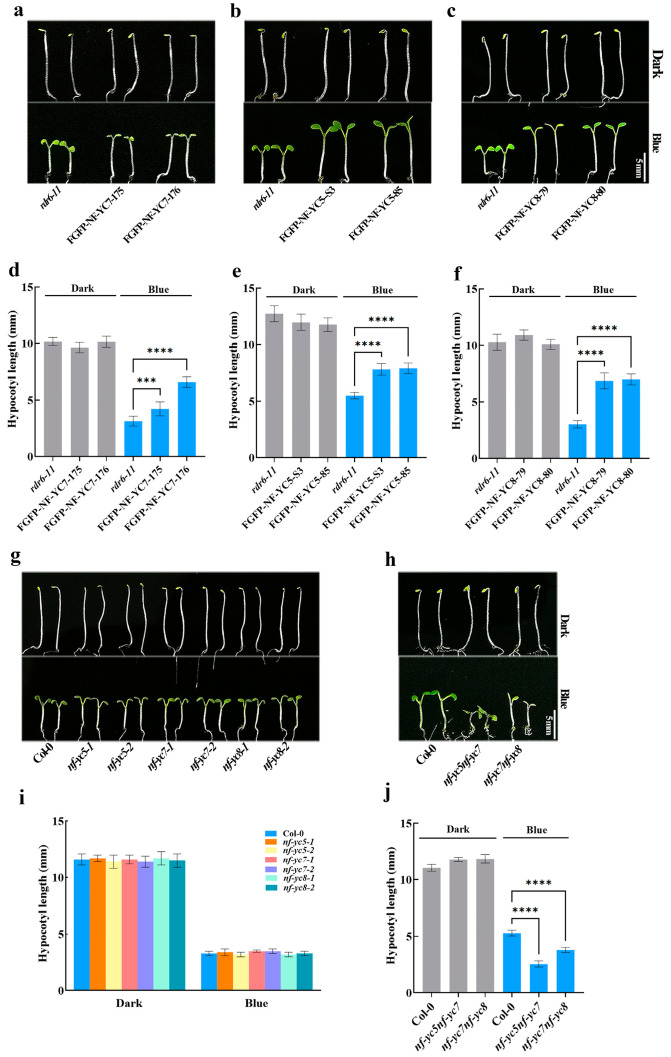
NF–YCs negatively regulate blue-light-mediated hypocotyl growth. (**a**–**c**) The representative hypocotyl images of the wild type (*rdr6-11*) and two independent NF–YCs–overexpressing lines; (**d**–**f**) hypocotyl lengths of the seedlings shown in (**a**–**c**); and (**g**,**h**) the representative hypocotyl images of the wild type (Col-0) and *NF*–*YCs* mutants. (**i**,**j**) Hypocotyl lengths of the seedlings shown in (**g**,**h**); all of the indicated seedlings grown under blue light (10 μmol m^−2^ s^−1^) or in the dark for 5 days were used to measure hypocotyl lengths (*** p < 0.001, **** p < 0.0001, compared with the wild type grown under blue light; one-way ANOVA; ±SD, *n* = 20). All experiments were repeated at least three times and the data from one repeat are shown.

To identify Arabidospis TFs involved in blue-light-mediated photomorphogenesis, we measured blue-light-induced hypocotyl lengths of TF–overexpressing lines, FGFP–TFs (FGFP: Flag and GFP–infused proteins), in the background of *rdr6*–*11*. We found that two independent NF–YC7–overexpressing lines, FGFP–NF–YC7–175 and FGFP–NF–YC7–176, showed longer hypocotyls than the wild type *rdr6*–*11* grown under blue light, but no differences from those grown in the dark (Figure 1a,d), and that FGFP–NF–YC7–infused proteins localized in hypocotyls (Figure S2). To investigate the role of two close NF–YC7 homologs, NF–YC5 and NF–YC8, in blue-light-mediated photomorphogenesis, we measured the hypocotyl lengths of their overexpression lines when grown in the dark and under blue light (Figure 1b,c,e,f). Based on the closer phylogenetic relationship among NF–YC7, NF–YC8, and NF–YC5, we measured the blue-light-mediated hypocotyl lengths of NF–YC5– and NF–YC8–overexpressing lines. Similarly, the hypocotyl lengths of NF–YC5– or NF–YC8–overexpressing lines—FGFP–NF–YC5–s3, FGFP–NF–YC5–85, FGFP–NF–YC8–79 and FGFP–NF–YC8–80 —were longer than those of the wild type *rdr6*–*11* grown under blue light but showed no difference from those grown in the dark, which means that NF–YC5, NF–YC7, and NF–YC8 may redundantly repress blue light-inhibited hypocotyl elongation. To further confirm this hypothesis, we employed *NF*–*YCs* single mutants (*nf*–*yc5*–*1*, *nf*–*yc5*–*2*, *nf*–*yc7*–*1*, *nf*–*yc7*–*2*, *nf*–*yc8*–*1, and nf*–*yc8*–*2*) and double mutants (*nf*–*yc5nf*–*yc7* and *nf*–*yc7nf*–*yc8*) and analyzed the hypocotyl lengths grown in the dark and under blue light (Figure 1g–j). The hypocotyl lengths of all the single mutants showed no difference from those of the wild type *Col-0* grown under both blue light and dark conditions (Figure 1g,i). As expected, the double mutants *nf*–*yc5nf*–*yc7* and *nf*–*yc7nf*–*yc8* developed shorter hypocotyls under blue light, similar to those of the wild type *Col*–*0* grown in the dark (Figure 1h,j). Consequently, NF–YC7, NF–YC5, and NF–YC8 redundantly and negatively regulate blue light-inhibited hypocotyl elongation.

### 2.2. Light Induces the Dynamical Expressions of NF–YCs in Hypocotyls

**Figure 2:**
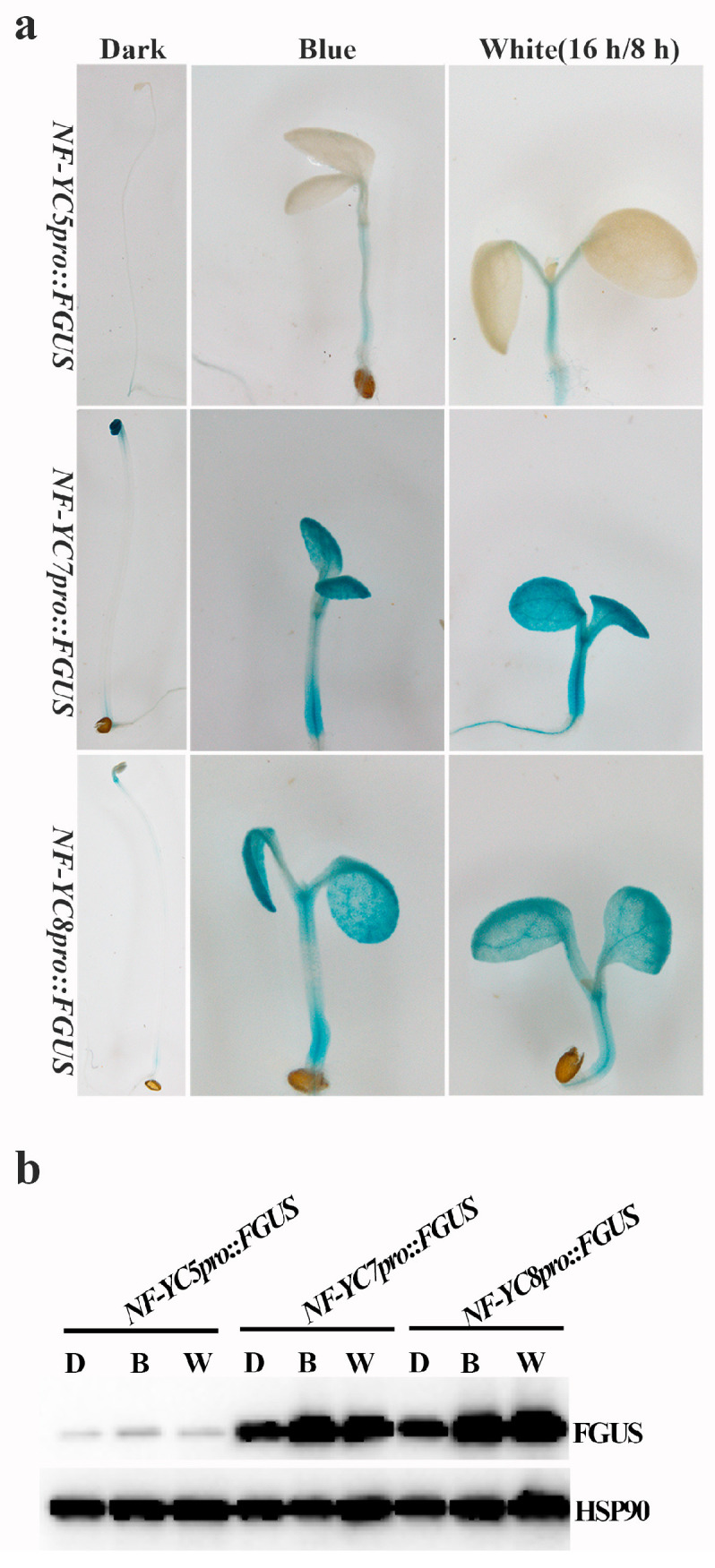
Light induces NF–YC expressions in hypocotyls. (**a**) GUS staining of the transgenic seedlings carrying *NF*–*YCspro::FGUS* grown in the dark or under continuous blue light (10 μmol m^−2^ s^−1^) or white light (16 h light/8 h dark) for 6 days, and (**b**) the expression levels of FGUS in the seedlings shown in (**a**); FGUS was probed by anti–Flag antibody. The loading control was probed by anti–HSP90.

As the repressors of blue light-inhibited hypocotyl elongation, the expressions of NF-YCs in hypocotyls may be regulated by light. To verify this assumption, we performed the GUS staining assay (Figure 2a). The results showed that the main localization of FGUS (FGUS: Flag and GUS-infused proteins) was in the apical or basal regions of hypocotyls when the transgenic seedlings carrying *NF*–*YCspro::FGUS *(*NF*–*YCspro*: *NF*–*YCs native promoter*) were grown in the dark. Clearly, FGUS accumulated in whole seedings, including hypocotyls, cotyledons, and roots when the seedlings were grown under continuous blue light (10 μmol m^−2^ s^−1^) or white light (16 h light/8 h dark). Moreover, we tested the FGUS expression in darkness, under blue light or white light using the Western blot (Figure 2b). In accordance with the above results, the expression of FGUS increased under blue light and white light, compared with that in darkness. All the results revealed that NF–YC promoters are light-responsive and light induced the accumulation of NF–YCs in whole seedlings. 

### 2.3. NF–YC7 Directly Interacts with CRY2

**Figure 3:**
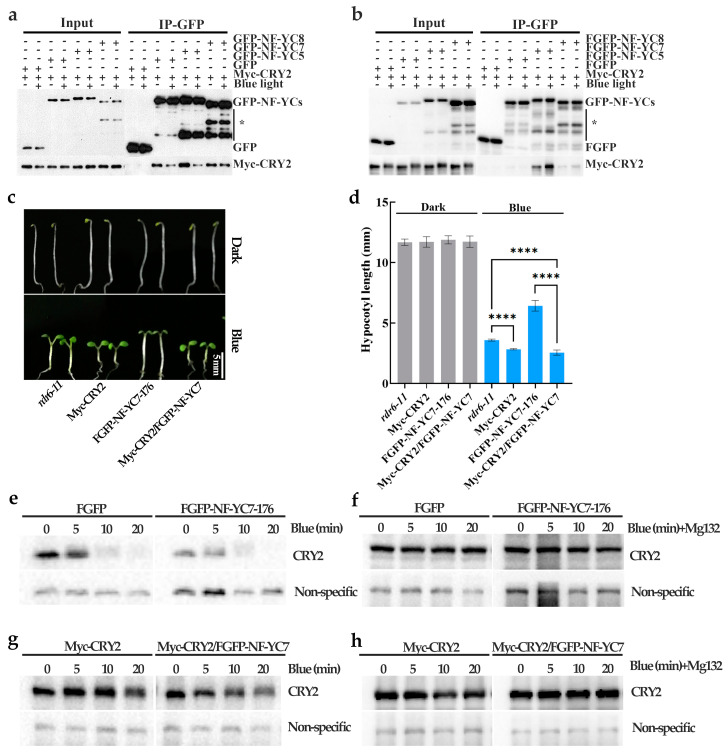
NF–YC7 negatively regulates CRY2-inhibited hypocotyl elongation. (**a**,**b**) The NF–YCs–CRY2 interactions in HEK293T cells (**a**) and *Arabidopsis* (**b**); protein extracts were immunoprecipitated by GFP agarose beads. The IP signal or the co-IP signal was probed by anti–GFP or anti–Myc antibodies, respectively. The star indicates non-specific bands. (**c**,**d**) The effect of NF–YC7 in CRY2–inhibited hypocotyl elongation. The representative hypocotyl images (**c**) and hypocotyl length (**d**) of the indicated genotypes grown in the dark or under blue light (10 μmol m^−2^ s^−1^) for 6 days (**** p < 0.0001, one-way ANOVA; ±SD, *n* = 20); all experiments were repeated at least three times, and the data from one repeat are shown. (**e**–**h**) NF–YC7 regulates the CRY2 degradation. (**e**,**f**) The endogenous CRY2 degradation in the indicated genotypes. (**g**,**h**) The degradation of CRY2 in the indicated genotypes. All of the indicated seedlings grown in the dark for 6 days were exposed to blue light (10 μmol m^−2^ s^−1^) for 0, 5, 10, and 20 min with (**e**,**g**) or without (**f**,**h**) MG132 incubation. CRY2 was detected using anti–CRY2 antibody. Non-specific bands were used as the loading control.

As reported, many light responsive proteins (such as CRY2, BIC1/2, HY5, etc.) participate in blue light-inhibited hypocotyl elongation [5,9,30]. To test whether NF–YCs interact with one of these light-responsive proteins, we employed co–IP assays in HEK293T cells and Arabidopsis. It was implied that NF–YC7 directly interacts with CRY2 (Figure S3). Therefore, we proposed that CRY2 may be the interacting protein of the NF–YC7 homologs, NF–YC5 and NF–YC8. Then, we verified the interactions between NF–YCs and CRY2 using two different assays. The results showed that Myc–CRY2 interacts with NF–YCs under both dark and-blue light conditions (Figure 3a,b). NF–YC7 had a stronger interaction with CRY2, compared with NF–YC5 and NF–YC8. 

### 2.4. NF–YC7 Negatively Regulates the CRY2–Mediated Hypocotyl Growth by Inactivating CRY2

Based on the NF–YC7–CRY2 interaction (Figure 3a,b) and their functions in blue-light-mediated hypocotyl growth, we assumed that NF–YC7 and CRY2 play a role in the same pathway. Therefore, we tested the genetic relationships between NF–YC7 and CRY2 in the blue-light inhibition of hypocotyl elongation. The hypocotyl lengths of the CRY2-overexpressing line (Myc–CRY2), the NF–YC7–overexpressing line (FGFP–NF–YC7–176), the co-overexpression line of CRY2 and NF–YC7 (Myc–CRY2/FGPF–NF–YC7), and the wild type (*rdr6-11*) were examined after growing in the dark and under blue light (Figure 3c,d). There were no differences among the tested seedlings grown in the dark. Blue light induced shorter hypocotyl lengths in Myc–CRY2 and Myc–CRY2/FGFP–NF–YC7 and longer hypocotyl in FGFP–NF–YC7–176, compared with the wild type. Myc–CRY2/FGFP–NF–YC7 developed similar hypocotyl lengths to Myc–CRY2 under blue light. This suggests that CRY2 and NF–YC7 regulate blue-light-inhibited hypocotyl elongation in the same pathway. CRY2 repressed NF–YC7–induced hypocotyl elongation under blue light, which indicates that CRY2 acts downstream of NF–YC7.

The hypocotyl lengths of the NF–YC7–overexpressing lines were similar to those of *cry2*–*1* grown under blue light and longer than those of the wild type. Therefore, we proposed that NF–YC7 might inactivate CRY2, and tested whether NF–YC7 affects the degradation of CRY2 in *Arabidopsis* (Figure 3e–h). Endogenous CRY2 began to degrade, not only in FGFP–NF–YC7–176, but also in control plants (FGFP–overexpressing line), because the etiolated seedlings grown in the dark for 6 days were exposed to blue light. Endogenous CRY2 degradation increased with the extension of blue-light treatment. However, endogenous CRY2 in FGFP–NF–YC7–176 degraded faster than in control plants (Figure 3e). Consistently, CRY2 degradation was faster in Myc–CRY2/FGFP–NF–YC7 than in Myc–CRY2 under blue light (Figure 3f). Because of the requirement of blue-light-dependent polyubiquitination for CRY2 degradation [12], we analyzed the effect of NF–YC7 on CRY2 degradation when pretreated with MG132 (a proteasome inhibitor that blocks the proteolytic activity of the 26S proteasome complex and inhibits protein degradation induced by the 26S proteasome). After incubation with MG132, blue light failed to induce CRY2 degradation in the presence or absence of NF-YC7 (Figure 3f,h). This suggested that NF-YC7 may increase the degradation of CRY2 through polyubiquitination. 

In summary, NF–YC7 suppresses CRY2-mediated hypocotyl growth by increasing CRY2 degradation.

### 2.5. NF–YCs Interact with CRY2, PIF4, and PIF5 

**Figure 4:**
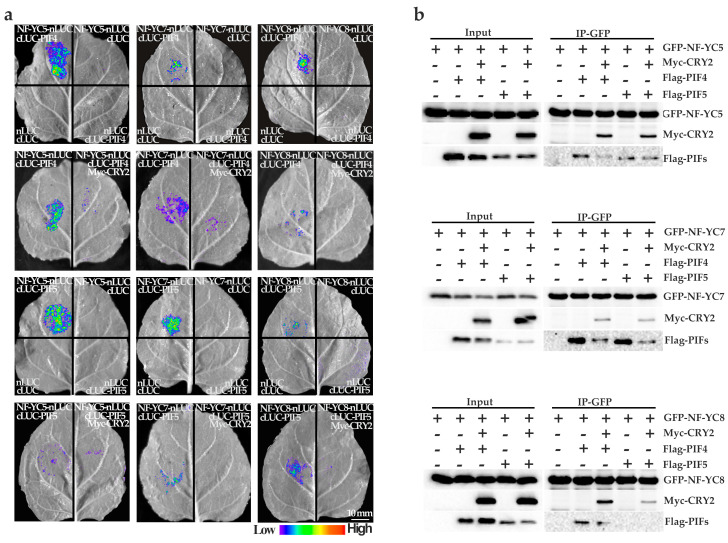
NF–YCs interact with CRY2 and PIF4/5. (**a**) The interactions between NF–YCs, CRY2, and PIF4/5 using the LCI assay in tobacco leaves (*N. benthamiana*). The LUC signal was visualized after incubation with Luciferin in the dark for 5 min. The exposure time was equal to 5 min; (**b**) the interactions between NF–YCs, CRY2, and PIFs using co–IP assay in HEK293T cells. GFP–NF–YCs were immunoprecipitated using GFP agarose beads. The IP signal (GFP–NF–YCs) and the co–IP signal (Myc–CRY2 and Flag–PIFs) were detected by anti–GFP, anti–Myc, and anti–Flag antibodies. The star indicates non-specific bands. Plus and minus signs mean that the candidate proteins are present and absent.

Based on the NF–YCs–CRY2 interactions (Figure 3a,b) in our study and the CRY2–PIF4/5 interactions previously reported [14], we hypothesized that NF–YCs, PIF4/5 and CRY2 might interact with each other. To examine the interactions, we carried out the LCI assay and co–IP assay. The results (Figure 4a,b) showed that CRY2 repressed NF–YC7–PIF4/5 interactions, while NF–YC8 did not clearly bind to PIF5 or PIF4 clearly. Interestingly, NF–YC5 bound to PIF5 with the same probability with or without CRY2, but bound to PIF4 similarly to NF–YC7.

### 2.6. PIF4 and PIF5 Act Downstream of NF–YCs

**Figure 5:**
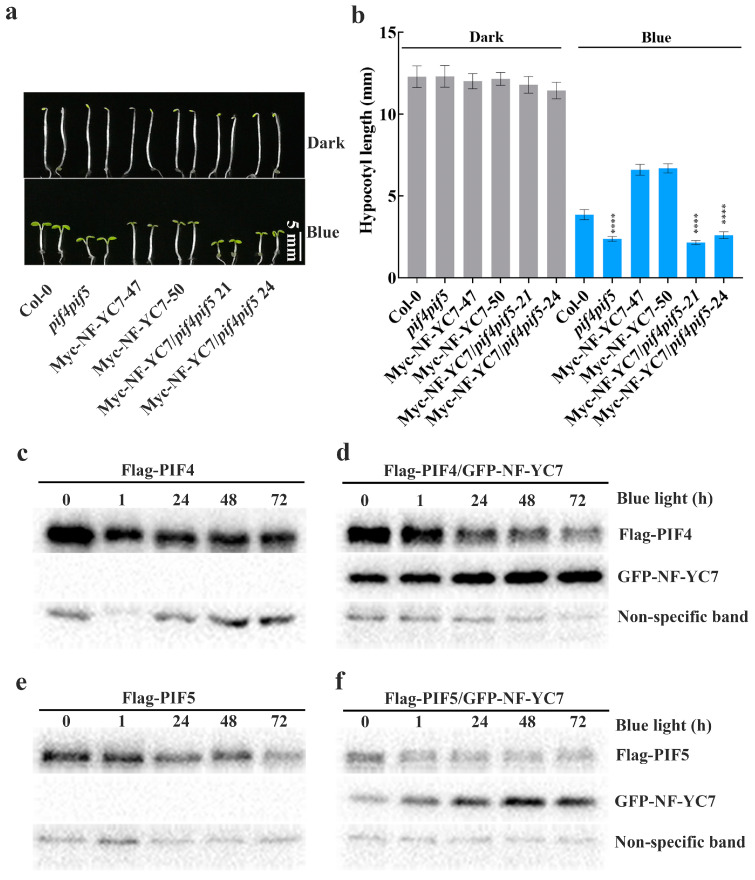
PIF4/5 act downstream of NF–YC7. (**a**,**b**) The representative hypocotyl images (**a**) and hypocotyl lengths (**b**) of all the indicated genotypes. All seedlings were grown under blue light (10 μmol m^−2^ s^−1^) or in the dark for 6 days (**** p < 0.0001, compared with the wild type grown under blue light; one-way ANOVA; ±SD, *n* = 20). All experiments were repeated at least three times and the data from one repeat are shown. (**c**–**f**) The effect of NF–YC7 on the activities of PIF4 (**c**,**d**) and PIF5 (**e**,**f**). Flag–PIF4/5: PIF4/5–overexpressing lines in the background of *rdr6*–*11*, Flag–PIF4/GFP–NF–YC7 and Flag–PIF5/GFP–NF–YC7: the co-overexpression lines of PIF4/5 and NF–YC7 in the background of *rdr6*–*11*. All the seedlings grown in the dark for 6 days were exposed to blue light (10 μmol m^−2^ s^−1^) for 0, 1, 12, 24, and 72 h and collected to verify the PIF4/5 activities using Western blot. GFP–NF–YC7 and Flag–PIF4/5 were detected by anti–GFP and anti–Flag antibodies, respectively. Non-specific bands were the loading controls.

PIF4 and PIF5 are responsible for blue-light-mediated hypocotyl growth as described previously [31] and in our study (Figure S5). To investigate whether PIF4/5 participate in NF–YC–regulated hypocotyl growth, we measured the hypocotyl lengths of the wild type *Col-0*, double mutant *pif4pif5*, two independent NF–YC7–overexpressing lines (Myc–NF–YC7–47 and Myc–NF–YC7–50), and two independent lines overexpressing NF-YC7 in *pif4pif5* (Myc–NF–YC7/*pif4pif5*–21 and Myc–NF–YC7/*pif4pif5*–24) after growing in the dark and under blue light (Figure 5c,d). After growing in the dark for 6 days, all of the above genotypes displayed similar hypocotyl lengths. Compared with *Col*–*0*, the NF–YC7–overexpressing lines developed longer hypocotyls, but *pif4pif5* and Myc–NF–YC7/*pif4pif5* had shorter hypocotyls after the treatment with blue light. The hypocotyl lengths of Myc–NF–YC7/*pif4pif5* were similar to those of *pif4pif5,* but shorter than those of *Col*–*0* grown under blue light. Conclusively, PIF4/5 act downstream of NF-YC7 to negatively regulate blue-light-inhibited hypocotyl elongation.

To verify whether NF–YC7 regulates PIF4/5, we examined the effect of NF–YC7 on the PIF4/5 protein levels using Flag–PIFs (PIF4/5–overexpressing lines) and Flag–PIFs/GFP–NF–YC7 (PIF4/5 and NF–YC7 co-overexpressing lines). PIF4 and PIF5 were slightly degraded because all seedlings grown in the dark for 6 days were exposed to blue light (Figure 5c,e). However, the PIF4/5 protein bands exhibited a slower mobility shift in Flag–PIFs/GFP–NF–YC7 compared with Flag–PIF4/5 under blue light (Figure 5d,f). This suggests that NF–YC7 may regulate PIF4/5 activities in blue light-inhibited hypocotyl elongation. 

## 3. Discussion

NF–YC5/7/8 repressing blue-light-inhibited hypocotyl elongation suggested that NF–YC5/7/8 should be expressed in hypocotyls. The GUS staining results (Figure 2a,b) indicated that light induces NF–YC7/5/8 accumulations in hypocotyls, but the expression of NF–YC7 was the strongest compared with that of NF–YC5 and NF–YC8. NF–YC5 had the weakest expression in hypocotyls under light treatment. This suggested that the promoter activities of NF–YC5/7/8 were different from each other, which may lead to their diverse functions. For example, NF–YC7 showed stronger binding with CRY2 than NF–YC5 and NF–YC8 (Figure 3a,b). However, the NF–YC5/7/8 expression patterns in this study were not consistent with those in a previous report [26]. Siefer et al. indicated that NF–YC7/5/8 did not localize in hypocotyls grown in the dark or under light using the GUS staining assay [26]. The possible reasons for this may be that the expressions of endogenous NF–YC7/5/8 were low or that they tested NF–YC expression patterns by employing the transgenic lines with a single insertion of *NF*–*YC7/5/8pro::GUS* (*NF*–*YC7/5/8pro*: *NF*–*YC5/7/8* native promoter) [26]. Contrarily, we chose 2–3 independent transgenic lines with high expressions of GUS driven by *NF*–*YC7/5/8* native promoters and detected GUS staining under dark, blue light, and white light conditions.

NF–YC1/3/4/9 and NF–YC7/5/8 belong to two different clades of the NF–YC family due to the differences in their amino acid sequences, as previously described [26]. Changes in the amino acids of NF–Y members may contribute to their unique functions [26]. This may be the reason why NF–YC1/3/4/9 [29,32] and NF–YC7/5/8 (Figure 1) have opposite functions in the blue-light inhibition of hypocotyl elongation. Moreover, NF–YC5 and NF–YC8 show stronger homologs to each other than to NF–YC7 because of the nonconservative changes in the required amino acids [26]. Our results (Figure 3a,b) also show that CRY2 preferentially interacts with NF–YC7, compared with NF–YC5 and NF–YC8, which provides functional evidence for the hypothesis of Siefer et al. [26]. 

CRY2 positively regulates blue-light-mediated hypocotyl growth [5]. CRY2 undergoes photoactivation and inactivation by interacting with many light-responsive proteins. PPKs (photoregulatory protein kinases 1, 2, 3, and 4) phosphorylate dimeric CRY2 to enhance CRY2 activity [8]. For CRY2 inactivation, photo-excited CRY2 is polyubiquitinated by LBR1/2 (Light-Response Bric-a-Brack/Tramtrack/Broad 1 and 2) and degraded by the 26S proteasome [11]. BIC1 (blue-light inhibitor of cryptochrome 1) interacts with CRY2 to suppress CRY2 dimerization and the interactions between CRY2 and its signaling partners [9]. In our study, NF–YC7, the CRY2–interacting partner, may promote CRY2 degradation via the 26S proteasome (Figure 3a,b,e–h). Additionally, our preliminary results showed that NF–YC5/7/8 preferentially interact with COP1 (constitutive photomorphogenic 1, CRY2–related E3 ligases), compared with another CRY2–related E3 ligases, LRB1/2 (Light-Response Bric-a-Brack/Tramtrack/Broad 1/2) (Figure S4). NF–YCs–E3 ligases interactions (Figure S4) and the light-induced accumulation of NF–YCs (Figure 2a,b) may provide a compete explanation for how NF–YCs recruit the indicated E3 ligases or enhance their activities to accelerate CRY2 degradation.

CRY2 directly interacts with PIF4/5 and modulates PIF activities in limiting blue-light-mediated photomorphogenesis [14]. In this study, we clarified that CRY2 represses the NF–YC7–PIF4/5 interactions and the NF–YC5–PIF4 interaction, but not the NF–YC5/8–PIF5 interactions and the NF–YC8–PIF4 interaction, to regulate photomorphogenesis (Figure 4). We proposed that NF–YC7 was in charge of interacting with CRY2 and PIF4/5. NF–YC5 may play a main role in recruiting PIF4.

Some known protein kinases, such as BIN2 (brassinosteroid-insensitive 2), can interact with NF–YC1/3/4/9 and phosphorylate PIF4 [29,33]. As reported, *FYPP1* and *FYPP3* (catalytic subunits of protein phosphatase 6) mutations produced lower mobility shifts of PIFs due to PIF3 and PIF4 phosphorylation [24]. Our results showed that the migrations of PIF4/5 were slower in the presence of NF–YC7 (Figure 5e,f). This suggests that NF–YC7 may maintain the phosphorylation statuses of PIF4/5 by activating some protein kinases or deactivating phosphatases involved in PIFs phosphorylation.

PIFs activities and stabilities are necessary for their functions. It was reported that *PIF4/5*–deficient mutants have no obvious hypocotyl growth compared with the wild type under limiting blue light [14], while the sustained activation of PIFs leads to hypocotyl over-elongation. Limiting blue light can keep the PIF4 protein constant but promotes the accumulation of PIF5, while high-intensity light can rapidly decrease the abundance of bHLH members, such as PIF1, PIF3, PIF4, and PIF5 [14,34]. Our study showed similar results to previous reports. Blue light (10 μmol m^−2^ s^−1^) led to the slight PIF4/5 degradation (Figure 5e,f) and induced shorter hypocotyl lengths of *pif4*, *pif5*, and *pif4/5* than that of the wild type (Figure S4). All of the above results provided evidence for the previous conclusion on light-intensity-dependent PIFs stabilization [35].

**Figure 6:**
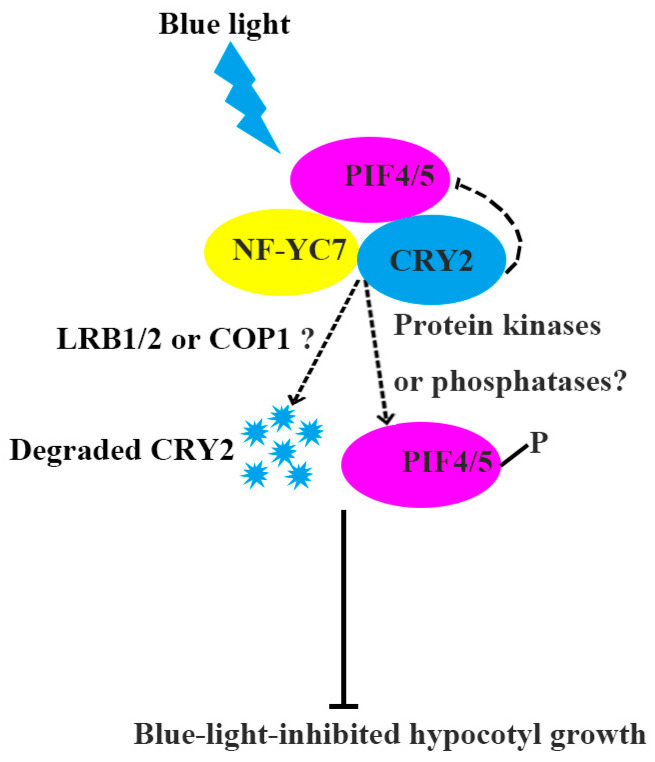
A hypothetical model depicting NF–YC7 repressing blue-light-inhibited hypocotyl growth.

In summary, we demonstrated the detailed mechanism of NF–YC7 suppressing blue-light-inhibited hypocotyl elongation (Figure 6). NF–YC7 physically interacts with CRY2 and PIF4/5. NF–YC7 may increase CRY2 degradation by recruiting or activating CRY2–related E3 ligases (COP1 or LRB1/2) or modulate PIF4/5 phosphorylation by activating some protein kinases or deactivating protein phosphatases. This study will be helpful for fully understanding the mechanism of blue-light-mediated photomorphogenesis.

## 4. Materials and Methods

### 4.1. Plant Materials

All the mutants used in this study were in the background of the Arabidopsis Columbia ecotype. Mutants *nf*–*yc5*–*1* (salk_130605), *nf*–*yc7*–*1* (salk_012179), *nf*–*yc8*–*1* (cs862600) and *nf*–*yc8*–*2* (salk_064020) were obtained from the ABRC (the Arabidopsis Biological Resource Center, https://www.arabidopsis.org, 1 January 2017). Mutants *pif4*, *pif5* and *pif4pif5* were kindly provided by Dr. Ying Li (Yangzhou University, Yangzhou, China). Mutants *nf*–*yc5*–*2* (inserted “A” at 455 bp) and *nf*–*yc7*–*2* (inserted “T” at 229 bp) were produced using CRISPR (clustered regularly interspaced short palindromic repeats, Figure S1) and confirmed by sequencing. To prepare the double mutant *nf*–*yc5nf*–*yc7*, *NF*–*YC5* was mutated in the background of *nf*–*yc7*–*1* using CRISPR because of the close physical locations of *NF*–*YC5* and *NF*–*YC7* in the same chromosome. The double mutant *nf*–*yc7nf*–*yc8* was generated by crossing *nf*–*yc7*–*1* and *nf-yc8*–*1*. 

To generate Arabidopsis TF-overexpressing lines (FGFP–TFs), Arabidopsis transcription factor candidates were cloned into the expression vector *pACT2::FGFPbar* (*pACT2*: *Actin 2* promoter; FGFP: Flag and GFP–infused proteins; *bar*: resistance gene) using LR recombination reaction (Invitrogen). To prepare the CRY2–overexpressing line Myc–CRY2, the coding sequences of *CRY2* amplified from Arabidopsis Columbia ecotypes were infused into *pACT2::cMYChyg* using In-Fusion Cloning methods. All of the above plasmids were transformed into *rdr6-11* by *the Agrobacterium tumefaciens*-mediated floral-dip method [36,37,38]. The plant expression vectors *pACT2::cMychyg* and *pACT2::FGFPbar* used in this study were modified from *pCambia3301*. All the primers used in this study are listed in Table S1. 

The co-overexpression line Myc–CRY2/FGFP–NF–YC7 was obtained by crossing Myc–CRY2 with FGFP–NF–YC7. To verify the genetic relationships between NF–YC7 and PIF4/5, the coding sequence of *NF*–*YC7* was infused into *pACT2::cMychyg* and introduced into *Col*–*0* and *pif4pif5,* respectively. 

### 4.2. Growth Conditions

Seeds were planted on MS medium, kept at 4 °C in the dark for 4 days, and then treated under white light for 1 day at 21 °C. After treatment using the above processes, seeds were grown in an intelligent growth chamber at 21 °C with different light treatments according to the experimental plans. The blue-light and green-light sources were obtained from Haibo Instrument and Equipment Company (Changzhou, China). The illumination meter used in this study was a TES–1339P (TES, Taipei, China).

### 4.3. Hypocotyl Length Measurement 

To measure the hypocotyl lengths, all the seeds were treated using continuous blue light (10 μmol m^−2^ s^−1^) or darkness for 6 days. At least 20 seedlings of each sample were measured using Image J 1.48 V. Data were analyzed using Excel 2016 and GraphPad Prime 9.0.0. The experiments were repeated at least three times and the results of one repeat are shown in this study. 

### 4.4. GUS Staining

To verify the expression patterns of *NF*–*YCs*, *NF*–*YCs* promoters (*NF*–*YCspro*) were amplified by high-fidelity PCR as previously described [26]. *NF*–*YCs* promoters were respectively infused into the 5′ terminal part of FGUS (Flag and GUS–infused protein) in the expression vector *pFGUSbar* derived from the binary vector *pCambia3301*. *NF*–*YCspro::FGUS* was transformed into *Col*–*0*. After treatment under dark, blue-light or long-day conditions (16 h light/8 h dark) for 6 days, the transgenic seedlings carrying *NF*–*YCspro::FGUS* were collected and incubated overnight in GUS staining buffer in the dark at 37 °C, and then washed using absolute ethanol until the chlorophyll totally disappeared, as previously described [39]. Pictures were taken using a Nikon SMZ18.

### 4.5. Degradation of CRY2

To verify the roles of NF–YCs in the CRY2 degradation, we employed two groups of genotypic materials. FGFP–NF–YC7 and the relative wild type (the transgenic line carrying *pACT2::FGFPbar*) were used to verify the effect of NF–YC7 on the endogenous CRY2 degradation. In addition, Myc–CRY2 and Myc–CRY2/FGFP–NF–YC7 were used to analyze the effect of NF–YC7 on CRY2 degradation. All of the above indicated seedlings grown in the dark for 6 days were exposed to blue light (10 μmol m^−2^ s^−1^) for 0, 5, 10, and 20 min with or without 50 μM MG132 (Selleck, Houston, TX, USA) incubation. Protein extraction and detection were performed as previously reported [11,40]. CRY2 were detected using anti–CRY2 antibody (Abcam, Cambridge, UK). 

### 4.6. Co–IP Assay

For the co–IP assay in *Arabidopsis*, Myc–CRY2/FGFP (the line co-overexpressing CRY2 and *pACT2::FGFPbar* empty vector) and Myc–CRY2/FGFP–NF–YCs were employed to verify the effects of light on the NF–YCs–CRY2 interactions in *Arabidopsis.* All the seedlings grown in the dark for 8 days were exposed to blue light (0, 5, 10, and 15 min) and then collected. Then, samples treated with blue light for 5, 10, and 15 min were mixed together to ensure that the duration of light treatment was sufficient and CRY2 was not yet fully degraded. The previously described protocol of protein extraction and purification was followed in this study, with minor modifications [11].

The co-IP assay was performed in HEK293T cells to identify in vitro protein interactions. To verify the NF–YCs–CRY2 interactions, GFP, GFP–NF–YCs, and Myc–CRY2 driven by the CMV5 promoter were expressed in HEK293T cells according to the experimental plans. To analyze the NF–YCs–CRY2–PIFs interactions, GFP–NF–YCs, Myc–CRY2 and Flag–PIFs driven by the CMV5 promoter were expressed in HEK293T cells according to the experimental plans. Cells carrying the indicated plasmids were treated using darkness or blue light (100 μmol m^−2^ s^−1^, 1 h) and then collected. The previously described protocols of HEK293T cells transformation and protein extractions were followed [11].

The protein complex was purified using GFP–Trap agarose beads (Chromotek, Munich, Germany) and separated by Western blot. Anti–GFP antibodies, anti–Myc antibodies, and anti-Flag antibodies (MBL, Tokyo, Japan) were used to detect FGFP/GFP–NF–YCs, Myc–CRY2 and Flag–PIFs, respectively.

### 4.7. Firefly Luciferase Complementation Imaging Assay (LCI)

For the LCI assay, the coding sequences of *NF*–*YCs* were fused into the N-terminal of nLUC and the coding sequences of *PIFs* were cloned into the C–terminal of cLUC, as previously described [41]. To verify the interactions between NF–YCs and PIFs, NF–YCs–nLUC or cLUC–PIFs were transiently expressed into *N. benthamiana*. To verify the effect of CRY2 on NF–YCs–PIF interactions, NF–YCs–nLUC and cLUC–PIFs were transiently expressed into *N. benthamiana*, in the absence or presence of Myc–CRY2. The protocol of transient expression was followed as previously described [42]. To detect LUC signals, the leaves were incubated with 1 mM luciferin in the dark for 5 min, and then images were captured using the In Vivo Plant Imaging System (Night Shade LB 985).

### 4.8. Dynamic Expressions of PIFs

To generate the PIF–overexpressing lines Flag–PIF4/5 and the co-overexpression lines GFP–NF–YC7/Flag–PIF4/5, the coding sequences of *NF*–*YC7*, *PIF4,* and *PIF5* were infused into *pACT2::GFPsul* or *pACT2::Flagbar* modified from *pCambia3301* and introduced into *rdr6*–*11*. To verify the expression dynamics of PIF4/5 regulated by NF–YC7, the transgenic seedlings grown in the dark for 6 days were exposed to blue light (10 μmol m^−2^ s^−1^) for 0, 1, 24, 48, and 72 h. Samples were collected and immediately stored in liquid nitrogen. Protein was extracted and separated by Western blot. Flag–PIF4 and Flag–PIF5 were detected using anti–Flag antibodies (MBL). Anti–GFP antibodies were used to detect GFP–NF–YC7. Non-specific bands were used as the loading control.

### 4.9. AGI Locus Numbers

RDR6 (AT3G49500), NF–YC5 (AT5G50490), NF–YC7 (AT5G50470), NF–YC8 (AT5G27910), CRY2 (AT1G04400), PIF4 (AT2G43010), and PIF5 (AT3G59060).

## Data Availability

Not applicable.

## Supplementary Materials

The following supporting information can be downloaded at: https://www.mdpi.com/article/10.3390/ijms241512444/s1.
