# Supplementary material for: Arabidopsis NF–YC7 Interacts with CRY2 and PIF4/5 to Repress Blue Light-Inhibited Hypocotyl Elongation
Source: Int J Mol Sci. 2023 Aug 4;24(15):12444. doi: 10.3390/ijms241512444 (PMC10419918; doi:10.3390/ijms241512444)
Supplement: Supplementary file 1 [file ijms-24-12444-s001.zip › ijms-2395574-supplementary.pdf]

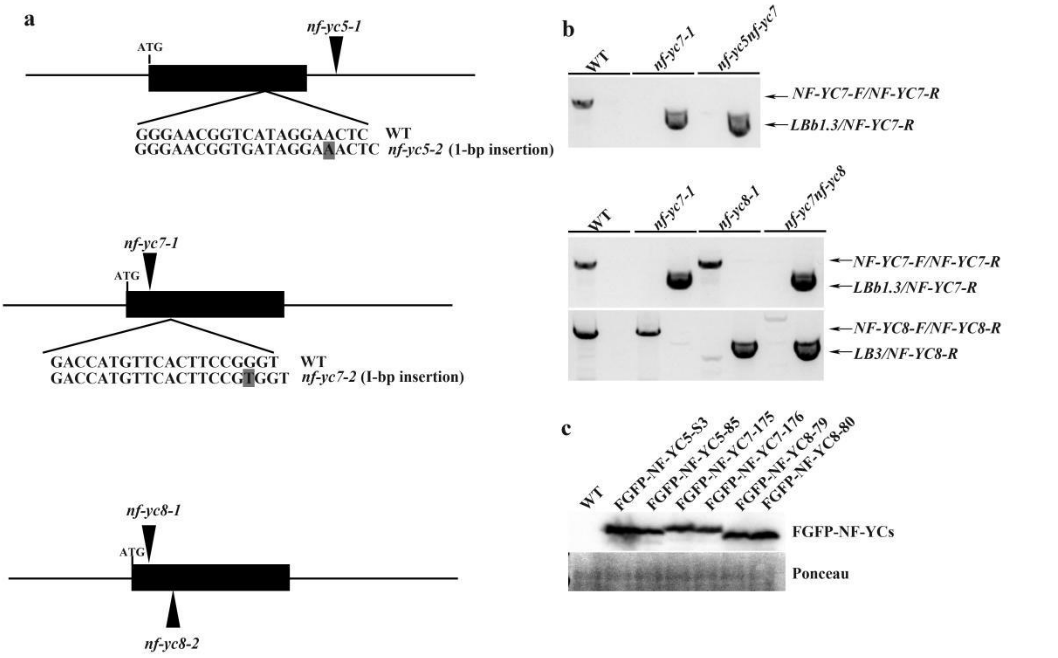

Figure S1. Information and identifications of *NF–YC*s–related genetic materials. (**a**) The schematic illustrations of *NF–YC* genes and the related mutants. WT: partial genomic sequence of native *NF–YC7*; *nf–yc5–2* and *nf–yc7–2*: the corresponding sequence of NF–YC5 or NF–*YC7* mutant carried 1-bp insertion by CRISPR; *nf–yc5–1, nf–yc7–1, nf–yc8–1* and *nf–yc8–2*: the T–DNA–inserted mutants from ABRC; Black box: Exon. Black line: 5′ or 3′ UTR of the indicated *NF–YC* genes. Triangle: the positions of each of the identified mutations. Gray box: the inserted nucleotide. (**b**) Identifications of the double mutant *nf–yc7nf–yc8* using PCR. Paired primers NF–YC–F+NF–YC–R for amplifying T–DNA, LB+NF–YC–R for amplifying genomic DNA. (**c**): Immunoblot analyses of protein levels in the wild type (WT) and the indicated NF–YCs–overexpressing lines. The FGFP–NF–YCs proteins were probed with anti–GFP antibodies. Ponceau was used as the loading control.

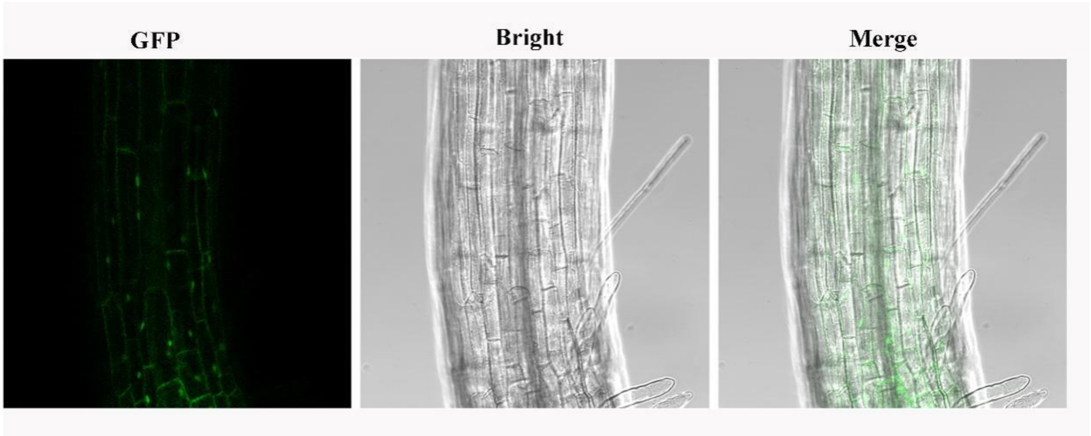

Figure S2. FGFP–NF–YC7 is localized in hypocotyl**.** The seedlings overexpressing FGFP–NF–YC7 driven by *ACT2 promoter* were grown under blue light for 6 days. And then, GFP fluorescence signals in hypocotyl were detected using Confocal laser scanning microscope (Zeiss 880). FGFP: short name for Flag and GFP–infused protein.

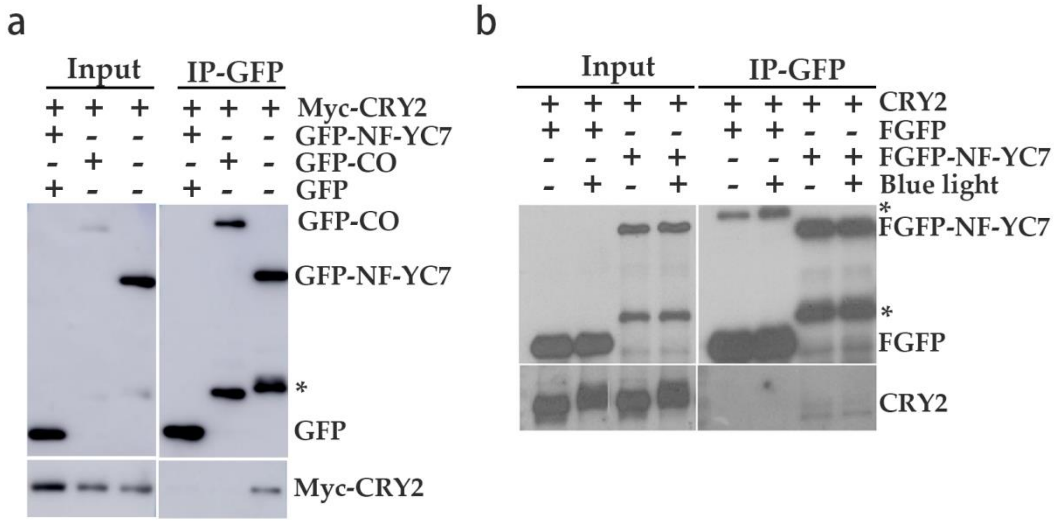

Figure S3. The interaction between NF–YC7 and CRY2**.** (a) The NF-YC7–CRY2 interaction using co-IP assay in HEK293T cells. GFP–NF–YC7, GFP–CO, and GFP were immunoprecipitated using GFP agarose beads. The HEK293T cells expressing the indicated proteins were treated under blue light (100 μmol m^{-2}

s^{-1}) for 1 h or in the dark and collected to check the CRY2–NF–YC7 interactions. CO: CONSTANS. The IP signal (GFP–NF–YC7, GFP–CO, and GFP) and the co–IP signal (Myc–CRY2) was detected by anti–GFP and anti–Myc antibodies.

(b) The NF–YC7–CRY2 interaction using co–IP assay in *Arabidopsis*. The seedlings expressing the indicated proteins were grown under blue light (10 μmol m^{-2} s^{-1}) or in the dark for 6 days and collected to check the CRY2–NF-YC7 interactions. Protein extracts were immunoprecipitated by GFP agarose beads. The IP signal (FGFP–NF–YC7 and FGFP) or the co-IP signal (CRY2) was probed by anti–GFP or anti–CRY2 antibodies.The star indicates non-specific bands. Plus and minus mean that the candidate proteins are present and absent.

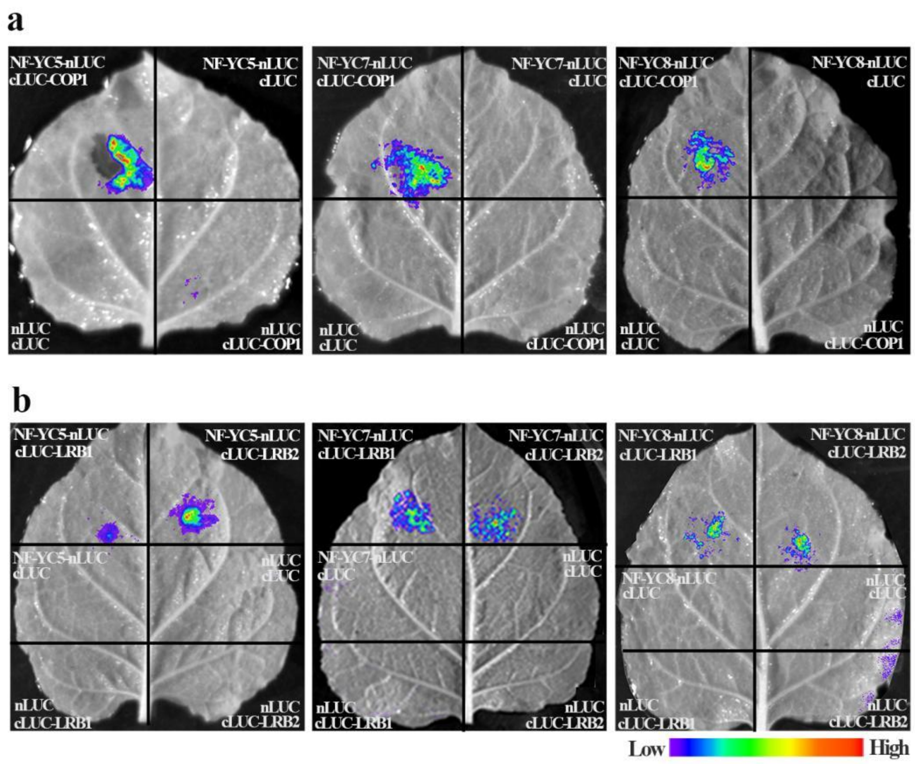

Figure S4. NF–YCs interact with CRY2–related E3 ligases. (**a**) The NF–YCs–COP1 interactions using LCI assay in *Tobacco*. The regions expressing NF–YCs–

nLUC and cLUC, nLUC and cLUC–COP1, nLUC and cLUC were used as negative controls. (**b**) The NF–YCs–LRB1/2 interactions using LCI assay in *Tobacco*. The regions expressing NF–YCs–nLUC and cLUC, nLUC and cLUC, nLUC and cLUC–LRB1, nLUC and cLUC–LRB2 were used as negative controls. LUC signal was visualized after incubating with 1 mM Luciferin for 5 min.

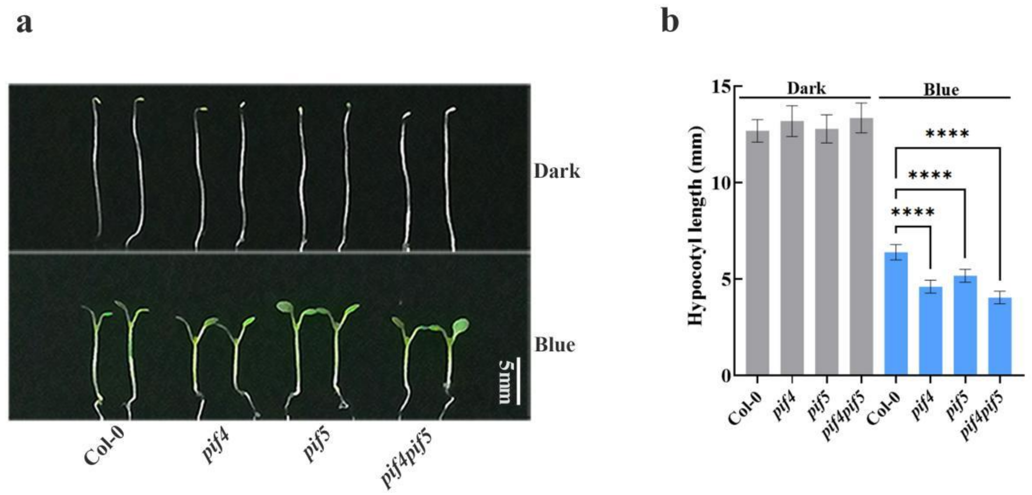

Figure S5. PIF4/5 modulate blue light-induced hypocotyl growth.

(a-**b**) The representative hypocotyl images (**a**) and hypocotyl lengths (**b**) of the indicated genotypes. All the seedlings were grown under blue light (10 μmol m^{-2} s^{-1}) or in the dark for 6 days (**** $p < 0.0001$, compared with the wild type grown under blue light; one-way ANOVA; ±SD, n = 20). All experiments were repeated at least three times and the data from one repeat are shown.

Table S1. Primers used in this study.

Primer names	Primer sequence(5'to3')	
GFP/FGFP–NF–YC5–F	TCCAGCTCCAGGATCCATGGAGAACAACAACAACAACCAC	For constructing overexpression lines
GFP/FGFP–NF–YC5–R	GAGAAAGCTTGGATCCTTAATTCCCACCGTTTCCTCC	

GFP/FGFP−NF−YC7−F	TCCAGCTCCAGGATCCATGGAAGAGAACAACGGCAACA	
GFP/FGFP−NF−YC7−R	GAGAAAGCTTGGATCCTCAATTACCGCCGCTGCTT	
GFP/FGFP−NF−YC8−F	TCCAGCTCCAGGATCCATGGAGAACAACAACGGCAAC	
GFP/FGFP−NF−YC8−R	GAGAAAGCTTGGATCCTTAGTTTCCGTCGTCACCTCCT	
Myc−NF−YC7−F	TGACCTCGAGACTAGTATGGAAGAGAACAACGGCAACA	
Myc−NF−YC7−R	GTCGCACCATACTAGTACCAGGAGGAGGGCCATTACCGCCGCTGCTTCCT	
FlagPIF4−F	TCCAGCTCCAGGATCCATGGAACACCAAGGTTGGAGTT	
Flag−PIF4−R	GAGAAAGCTTGGATCCCTAGTGGTCCAAACGAGAACCG	
Flag−PIF5−F	TCCAGCTCCAGGATCCATGGAACAAGTGTTTGCTGATTG	
Flag−PIF5−R	GAGAAAGCTTGGATCCTCAGCCTATTTTACCCATATGAAGA	
CRY2−cMyc−F	TGACCTCGAGACTAGTATGAAGATGGACAAAAAGACTATAG	
CRY2−cMyc−R	GTCGCACCATACTAGTACCAGGAGGAGGGCCTTTGCAACCATTTTTTCCC	
NF−YC5−BamHI−nLUC−F	TCGGTACCCGGGATCCTTATGGAGAACAACAACAACAACCAC	For LCI Assay
NF−YC5−SalI−nLUC−R	ACGAGATCTGGTCGACATTCCCACCGTTTCCTCCAT	
NF-YC7−BamHI−nLUC−F	TCGGTACCCGGGATCCTTATGGAAGAGAACAACGGCAACA	
NF−YC7−SalI-nLUC−R	ACGAGATCTGGTCGACATTACCGCCGCTGCTTCCT	
NF−YC8−BamHI−nLUC−F	TCGGTACCCGGGATCCTTATGGAGAACAACAACGGCAAC	
NF−YC8−SalI−nLUC−R	ACGAGATCTGGTCGACGTTTCCGTCGTCACCTCCTTT	
cLUC−BamHI−COP1−F	GCGGTACCCGGGATCCTATGGAAGAGATTTCGACGGATC	
cLUC−BamHI−COP1−R	TCCATTTGTTGGATCCGTCACGCAGCGAGTACCAGAAC	

cLUC−BamHI−LRB1−F	GCGGTACCCGGGATCCTATGAGAGGTTCCAATAACACCGA	
cLUC−BamHI−LRB1−R	TCCATTTGTTGGATCCGTCAGTGCAGGTCTGAGGAACG	
cLUC−BamHI−LRB2−F	GCGGTACCCGGGATCCTATGAGAGGTACTACTGAGAATACGGATC	
cLUC−BamHI−LRB2−R	TCCATTTGTTGGATCCGCTAAGGATCTGTAGACCTTTTGATGG	
cLUC−BamHI−PIF4−F	GCGGTACCCGGGATCCTATGGAACACCAAGGTTGGAGTT	
cLUC−BamHI−PIF4−R	TCCATTTGTTGGATCCGCTAGTGGTCCAAACGAGAACCG	
cLUC−BamHI−PIF5−F	GCGGTACCCGGGATCCTATGGAACAAGTGTTTGCTGATTG	
cLUC−BamHI−PIF5−R	TCCATTTGTTGGATCCGTCAGCCTATTTTACCCATATGAAGA	
NF−YC5pro::FGUS−F	ATGATTACGAATTCGAGCTCGATTTCGCCAAATAGGCTTTGA	For GUS staining assay
NF−YC5pro::FGUS−R	TGTAGTCCATACTAGTTTTTTTTTGTTTGAAGTGTTTGGG	
NF−YC7pro::FGUS−F	ATGATTACGAATTCGAGCTCATCCGCTTTAGGTGACCAGTT	
NF−YC7pro::FGUS−R	TGTAGTCCATACTAGTTTTGATCTTTACGTTTTTCTTGTTC	
NF−YC8pro::FGUS−F	ATGATTACGAATTCGAGCTCTTGCTCGGTGTCTGATACG	
NF-YC8pro::FGUS-R	TGTAGTCCATACTAGTAAGATTTTTTTCTTTTTTGAAGTTTC	
pQCMV−GFP−NF−YC5−F	CTCCAGCTCCAGCTGGTACCATGGAGAACAACAACAACAACCAC	For co-IP Assay in HEK293T cells
pQCMV−GFP−NF−YC5−R	TAAGCGTGCTCAGCGGTACCTTAATTCCCACCGTTTCCTCC	
pQCMV−GFP−NF−YC7−F	CTCCAGCTCCAGCTGGTACCATGGAAGAGAACAACGGCAACA	
pQCMV−GFP−NF−YC7−R	TAAGCGTGCTCAGCGGTACCTCAATTACCGCCGCTGCTT	
pQCMV−GFP−NF−YC8−F	CTCCAGCTCCAGCTGGTACCATGGAGAACAACAACGGCAAC	

pQCMV−GFP−NF−YC8−R	TAAGCGTGCTCAGCGGTACCTTAGTTTCCGTCGTCACCTCCT	
pQCMV−Flag−PIF4−F	CGACAAGGCTACTAGTATGGAACACCAAGGTTGGAGTT	
pQCMV−Flag−PIF4−R	TAAGCGTGCTCAGCGGTACCCTAGTGGTCCAAACGAGAACCG	
pQCMV−Flag−PIF5−F	CGACAAGGCTACTAGTATGGAACAAGTGTTTGCTGATTG	
pQCMV−Flag−PIF5−R	TAAGCGTGCTCAGCGGTACCTCAGCCTATTTTACCCATATGAAGA	
pCMV−Myc−CRY2−F	GGAGGACCTGGGATCCATGAAGATGGACAAAAAGACTATAG	
pCMV−Myc−CRY2−R	TAGCAGGCCTGGATCCTAAGCGTGCTCAGCGGTACC	
NF−YC7−sgRNA−F	ATTGGACCATGTTCACTTCCGGGT	For generating CRISPR lines
NF−YC7−sgRNA−R	AAACACCCGGAAGTGAACATGGTC	
NF−YC5−sgRNA−F	GATTGGGAACGGTGATAGGAACTC	
NF−YC5−sgRNA−R	AAACGAGTTCCTATCACCGTTCCC	
nf−yc5−1−F	TGTGCGCCAAATTCCGTT	For identifying T-DNA inserted mutants
nf−yc5−1−R	GATTTCTTGCGTGATGATGTCC	
nf−yc5−2−F	TGTGCGCCAAATTCCGTT	
nf−yc5−2−R	GATTTCTTGCGTGATGATGTCC	
nf−yc7−1−F	CATTGACGACATTCCTCATATGC	
nf−yc7−1−R	ATTTCCCCAGCTGCCTCCT	
nf−yc8−1−F	CGGTTTAAGTGAATTGCTGCG	
nf−yc8−1−R	GCGACAAGAACGAGAAGAAGGT	
nf−yc8−2−F	TATTAGTTTCCGTCGTCACCTCC	
nf−yc8−2−R	TGAGCCGTGTGACTGTAGAAGG	
LBb1.3	ATTTTGCCGATTTCGGAAC	
LBb3	TAGCATCTGAATTTCATAACCAATCTCGATACAC	
